# A Systematic Review of Human Amnion Enhanced Cartilage Regeneration in Full-Thickness Cartilage Defects

**DOI:** 10.3390/biomimetics9070383

**Published:** 2024-06-25

**Authors:** Nur Farah Anis Abd Halim, Atiqah Ab Aziz, Sik-Loo Tan, Veenesh Selvaratnam, Tunku Kamarul

**Affiliations:** 1 Tissue Engineering Group (TEG), National Orthopaedic Centre of Excellence for Research and Learning (NOCERAL), Department of Orthopaedic Surgery, Faculty of Medicine, Universiti Malaya, Kuala Lumpur 50603, Malaysia; eyqa@um.edu.my (A.A.A.); tansikloo@gmail.com (S.-L.T.); 2 Joint Reconstruction Unit (JRU), National Orthopaedic Centre of Excellence for Research and Learning (NOCERAL), Department of Orthopaedic Surgery, Faculty of Medicine, Universiti Malaya, Kuala Lumpur 50603, Malaysia; veenesh_selvaratnam@yahoo.co.uk

**Keywords:** human amnion, cartilage regeneration, cartilage defect, orthopedics

## Abstract

Cartilage defects present a significant challenge in orthopedic medicine, often leading to pain and functional impairment. To address this, human amnion, a naturally derived biomaterial, has gained attention for its potential in enhancing cartilage regeneration. This systematic review aims to evaluate the efficacy of human amnion in enhancing cartilage regeneration for full-thickness cartilage defects. An electronic search was conducted on MEDLINE-PubMed, Web of Science (WoS), and the Scopus database up to 27 December 2023 from 2007. A total of 401 articles were identified. After removing 125 duplicates and excluding 271 articles based on predetermined criteria, only 5 articles remained eligible for inclusion in this systematic review. All five eligible articles conducted in vivo studies utilizing rabbits as subjects. Furthermore, analysis of the literature reveals an increasing trend in the frequency of utilizing human amnion for the treatment of cartilage defects. Various forms of human amnion were utilized either alone or seeded with cells prior to implantation. Histological assessments and macroscopic observations indicated usage of human amnion improved cartilage repair outcomes. All studies highlighted the positive results despite using different forms of amnion tissues. This systematic review underscores the promising role of human amnion as a viable option for enhancing cartilage regeneration in full-thickness cartilage defects, thus offering valuable insights for future research and clinical applications in orthopedic tissue engineering.

## 1. Introduction

Cartilage defects, which include damage of both chondral bone and articular cartilage, are attributed to various conditions such as trauma, aging, and inflammation. Previous studies have indicated that full-thickness chondral defects or osteochondral defects can lead to articular cartilage loss and the development of early osteoarthritis [1,2]. Numerous surgical interventions have been proposed for addressing these defects, including microfracture, autograft, or allograft osteochondral transplantation, as well as cell transplantation, which involves either stem cells or chondrocytes. However, the repair of cartilage defects in humans can be a difficult procedure [3]. Although these treatments have yielded favorable results, there are cases of undesirable complications and occasional failure [4].

Thus, cartilage tissue engineering approaches have emerged with the aim to repair joint defects and restore articular function via biomaterials used in combination with multipotent cells either associated with or without bioactive molecules [4], eliminating or at least delaying the need for joint replacement. Such minimally invasive therapy combines the use of chondrocytes and biomaterials. Recently, there has been a significant acceleration in the search for and development of new and appropriate biomaterials for tissue engineering. This is particularly true in the exploration of natural biomaterials, which have been discovered to mimic the biological and mechanical functions of the native extracellular matrix (ECM) present in various tissues within the body [5].

Human amniotic membrane (HAM) is a naturally derived biomaterial which has been used for decades in regenerative medicine applications. The innermost layer of the HAM is composed of three different layers: the basement membrane, a single layer of epithelial cells, and an avascular collagenous stroma which further consists of three contiguous but distinct layers, the inner compact layer, middle fibroblast layer, and the outermost spongy layer [6]. The basement membrane is made up of type IV, V, and VII collagen in addition to fibronectin and laminin and serves as one of the thickest membranes in the human body which can withstand the cryopreservation technique [7].

HAM is abundant in ECM components, playing a crucial role in tissue reconstruction and organ formation due to its positive impact on cell survival, migration, proliferation, and differentiation. The ECM not only provides mechanical support but also introduces various biophysical and biochemical stimuli. For example, in cartilage, ECM offers nutrients and mechanical support for chondrocytes. Given the unique features and advantages of HAM, it has found extensive applications in both clinical practice and fundamental research. The HAM has been shown to have anti-inflammatory, anti-fibrotic, anti-angiogenic as well as anti-microbial properties [6].

In the latter part of the 20th century, HAM emerged as one of the pioneering biomaterials employed in the development of tissue-engineered constructs that support cell migration and the growth of new tissue [8]. HAM has grown to involve multiple fields of surgery and medicine in the past 50 years [9]. Notably, since 1995, there has been an increasing number of reports detailing its application in tissue engineering (TE), particularly as a biomaterial in soft tissue engineering within the realms of dermatology, plastic surgery, skin transplantation, and as a biological dressing for ophthalmic healing. Additionally, HAM is gaining momentum as an innovative material in the cardiac field, attributed to its exceptional properties as a scaffold for blood vessels and as a substitute for the pericardium. Apart from that, the rich structure of HAM supports its use as a natural biomaterial for clinical applications for cartilage repair [8].

HAM, being a readily available biocompatible material, also has some challenges due to mechanical stability issues, variations between donors, and alterations in the membrane properties associated with preservation method [7]. These challenges may potentially lead to complications during its utilization, including the risk of bacterial, viral, or fungal infections. Additionally, secondary complications linked to inflammation may arise during the application of HAM. Despite the reports about HAM’s anti-inflammatory properties, some research groups have indicated a minor inflammatory response in histological sections from wounds treated with HAM grafts. Presently, there are 130 review articles available on HAM as indicated by the existing data. However, as of now, there is no study that summarizes the techniques of utilizing HAM for cartilage regeneration based on amniotic cell regenerative therapies. Therefore, this systematic literature review and meta-analysis was performed and aimed to determine the efficacy of human amnion for full-thickness cartilage repair and enhancing cartilage regeneration.

## 2. Materials and Methods

This systematic review was performed according to the Preferred Reporting Items for Systematic Reviews and Meta-Analyses [10], Registration ID – 555307.

### 2.1. Search Strategy

An electronic search was conducted on MEDLINE-PubMed, Web of Science (WoS), and Scopus databases, for articles published in English, up to 27 December 2023 from 2007 to increase the chances of extracting more related articles. The reference lists of all publications selected were manually screened and additional articles complemented the electronic search. The search strategy for each database is shown in Table 1. The search strategy was conducted by finding the title, abstract, and keywords related to keywords chosen for this study.

### 2.2. Selection Criteria

All in vivo pre-clinical and clinical studies involving the HAM cells for cartilage regeneration were included. Only studies published in English with their abstracts available on the database were considered. In vitro studies only; proceedings articles and systematic reviews were excluded.

### 2.3. Search Outcomes

**Figure 1:**
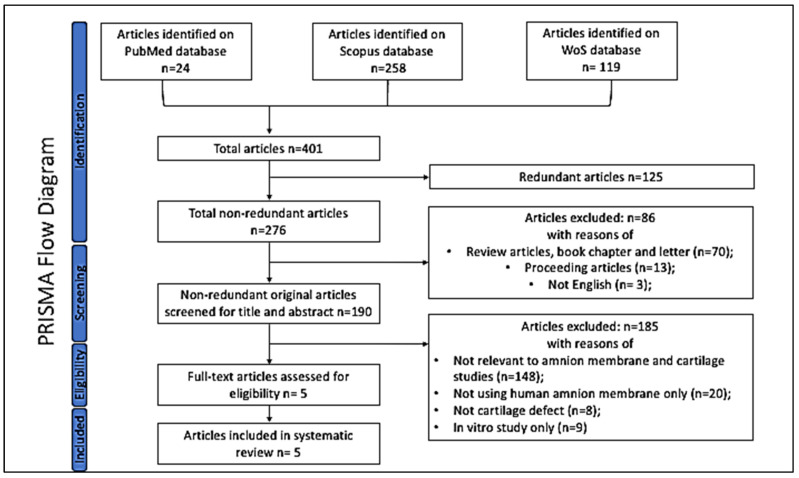
PRISMA (Preferred Reporting Items for Reviews and Meta-Analyses) workflow for the systemic selection process for articles. Flow chart for the literature search presenting the number of articles identified through the databases.

The search terms used for exhaustive searches against the three databases were summarized in Figure 1. The articles identified by the searches were compiled and subjected to an initial screening based on their title and/or abstract. The systematic search initially identified 401 articles over a 10-year period, reflecting a broad interest in the use of human amniotic membrane in various medical applications. However, a total of 190 non-redundant original articles were identified for detailed screening. The inclusion criteria were original research articles related to human amnion membrane for full-thickness cartilage defects and published in English. Exclusion criteria included non-original articles (review articles, book chapters, letters, and proceedings articles), articles not published in English, those not relevant to amnion membrane and cartilage studies, those not using amnion membrane exclusively, studies not addressing cartilage defects, and those reporting solely in vitro studies of human amnion membrane.

The majority of the initially identified articles did not meet these specific criteria. Many articles utilized human amniotic membrane but did not focus on its efficacy for full-thickness cartilage repair and cartilage regeneration, which is the core of the research question. Numerous studies were unrelated to cartilage studies, investigating other medical applications of the amniotic membrane and thus not providing relevant data for the review. Additionally, some studies did not use the amniotic membrane as the sole intervention, making it difficult to isolate its specific effects on cartilage repair. Several articles reported solely in vitro studies of human amniotic membrane or other contexts that were not pertinent to the research focus on in vivo cartilage defect models.

Based on these stringent inclusion and exclusion criteria, a total of five articles were eligible for this systematic review. The careful determination of inclusion and exclusion criteria is crucial, ensuring a focused, distinctive selection and preventing bias in the study and literature research selection process. While this resulted in a smaller number of included studies, it enhanced the validity of the conclusions by focusing on high-quality, directly relevant research. The five selected articles represent the most pertinent and rigorous studies available that address the specific research question. Drawing valid conclusions about the effectiveness of human amniotic membrane for cartilage repair requires high-quality evidence from studies that directly investigate this application. Including studies that do not meet these criteria would dilute the findings and potentially lead to inaccurate conclusions. The aim was to provide a focused and reliable synthesis of the best available evidence, ensuring the relevance and quality of the conclusions.

## 3. Results

A full list of the selected original articles is reported in Table 2. From five of the studies, two of them used HAM as a scaffold to repair osteochondral defects, while three of them seeded HAM with cells.

### 3.1. Use of HAM to Enhance Cartilage Regeneration for Repairing a Full-Thickness Cartilage Defect

All the studies investigating the use of HAM to enhance cartilage regeneration for repairing a full-thickness cartilage defect procured HAM from humans and transplanted it into rabbits. Although human joints differ from those of rabbits in several anatomical and physiological aspects, rabbit models are extensively used in preclinical studies of cartilage repair and regenerative medicine [16]. Numerous in vivo studies have shown that small and large animal models can benefit clinical conditions by assessing the efficacy and safety of new therapeutic interventions, devices, and biomaterials in animals with similar diseases/defects to humans [17]. Additionally, rabbits are an appropriate choice due to their manageable size, ease of handling, and the well-established surgical techniques available for creating and evaluating cartilage defects [18].

HAM was either applied on the defect or inserted into the cartilage defect. Additionally, HAM was used in various forms, including fresh (*n* = 4), decellularization (*n* = 1), and de-epithelialization (*n* = 1). HAM was seeded with cells before being implanted in the animal models (Table 3).

All of the studies assessed the efficacy of HAM when used alone to regenerate cartilage (Table 3). Covering the defect with HAM significantly enhanced cartilage repair. HAM scaffolds are natural biological materials that play important roles in osteochondral repair and cartilage regeneration [19]. Ideal cartilage repair aims to restore key properties of the original hyaline cartilage in terms of histological structure and biomechanical functions, which can only be achieved by replacing it with healthy cartilage tissue [20]. Components such as hyaluronan acid, proteoglycans, and collagens have been found between the ECM of HAM and native cartilage. According to Fenelon et al. (2021), the potential of fresh, cryopreserved, lyophilized, or dried amnion to act as MSCs or chondrocytes carriers and promote chondrogenic differentiation has been investigated with success [21].

### 3.2. Isolation of and Cultivation of MSCs

MSCs can be isolated using different methods as summarized in Table 4. The most commonly used approach in the articles reviewed for this study was isolating human MSCs from the amnion of human placentas (*n* = 3). Isolating MSCs from different parts of the placenta may result in variations in proliferation and differentiation potentials, owing to complex structures and functionalities of the placenta [22]. Another widely used method for isolating MSCs is the bone marrow culture method. Bone marrow is the most common source of MSCs which has been successfully isolated and characterized from many species, including pigs, rats, rabbits, dogs, sheep, mice, and humans [23]. One study used articular cartilage harvested from the knee joints of rabbits.

The culture condition used in studies is LG-DMEM/F12 medium supplemented with 10% foetal bovine serum (FBS). This medium is widely used as a basal medium to support the growth of many different mammalian cells. FBS is a common component of animal cell culture media used as a supplement, which aids in supplying hormone factors for cell proliferation and growth [24]. Penicillin/streptomycin solution has been used as an antibiotic solution for the culture of mammalian cells to control bacterial contamination. In all studies, the third generation of cells were used for osteogenic and adipogenic differentiation. The third generation of MSCs had the highest proliferative activity. One study observed the cell morphology, adhesion to plastic, as well as the expression of markers [25]. Researchers mentioned that MSCs lack unique cell membrane surface markers and can be identified by three biological characteristics: adherent properties, a series of cell-specific expression of cell surface markers, and multilineage differentiation potential [25,26].

### 3.3. Surgical Procedure and Creation of Osteochondral Defects

The animal model has been created with osteochondral defects located at the femoral condyle for all the studies, with different sizes of defects created analogous in the studies, with a diameter ranging from 3 mm to 5 mm and a depth of 3 mm. Four studies investigated the potential of using HAM seeded with MSCs (Table 5), which showed seeded HAM displayed chondrogenic potential as it demonstrated regenerative properties on the osteochondral defects made. HAM is an abundant tissue that may be an important source for transplanted chondrocytes in cartilage regeneration in vivo [27]. Two studies assessed the potential of the decellularized/de-epithelialized HAM seeded with MSCs to promote cartilage regeneration (Table 4), and they all reported that seeded HAM provided a dominant effect on cartilage defect repair, as the defect area was filled with newly formed cartilage.

The effectiveness of various treatments for osteochondral defects was evaluated, taking into consideration the size of the defects. When analyzing small defects (diameter < 4 mm), it was observed that the stromal side of denuded human amniotic membrane (HAM), referred to as DHS, demonstrated superior outcomes compared to other treatments [28]. In the DHS group, the defect area was completely filled with mature cartilage at 8 weeks post-surgery, indicating robust cartilage regeneration. The stromal side of denuded HAM contains abundant cartilage extracellular matrix (ECM) molecules, such as hyaluronic acid, which may facilitate cartilage formation from embryonic mesenchymal progenitor cells. Additionally, the three-dimensional environment provided by the stromal side promotes the production of ECM by seeded chondrocytes, further supporting cartilage regeneration.

In contrast, when evaluating small defects treated solely with fibrin glue, only minimal tissue regeneration was observed, with weak histological staining indicating slight degeneration at the defect edges [27]. This suggests limited effectiveness of fibrin glue alone in promoting cartilage repair in small defects.

For large defects (diameter ≥ 4 mm), the use of HAM sheets encapsulating cartilage particles, whether induced or seeded with chondrocyte cells, demonstrated promising outcomes across all studies [29]. Macroscopic observations indicated successful repair, with the defect area almost completely filled with a white semitranslucent tissue, and histological analysis revealed characteristics consistent with cartilage regeneration. These findings suggest that HAM-based scaffolds have the potential to effectively repair large osteochondral defects by promoting cartilage and bone regeneration within the defect site.

The comparison of treatment outcomes between small and large defects underscores the importance of tailoring treatment approaches based on defect size. While small defects may benefit from interventions that provide a supportive matrix and facilitate cell-mediated repair, such as DHS, larger defects may require more complex scaffolds, like HAM encapsulating cartilage particles, to achieve optimal regeneration outcomes. These findings have significant implications for clinical practice, emphasizing the need for personalized treatment strategies tailored to the specific characteristics of the osteochondral defect.

### 3.4. Histological Examination

The table summarizes histological examination methods by various studies along with their respective results (Table 6). Three of the studies fixed the samples in 4% paraformaldehyde for 24 h, decalcified for approximately 2 weeks, and embedded in paraffin. One of the studies prepared the specimen by fixation in 10% buffered formalin for 48 h and embedded in paraffin, while the other studies did not mention the specimen preparations for histological assessment. Fixation involves the use of chemicals to maintain the natural structure of tissue and safeguard it from degradation by forming irreversible cross-links between proteins. Another effective fixative is paraffin–formalin, where its advantage lies in being the preferred fixative for immunostaining [30].

One study conducted sagittal sections with a thickness of 4 μm, while another study did not specify the thickness of sagittal sections. Three other studies were sectioned to a thickness of 5 μm. All of the studies employed the hematoxylin and eosin (H&E) staining method, while some of the studies had Safranin O/Fast Green (SO/FG) and toluidine blue (TB) as their additional staining methods. The H&E staining method is widely used in histology for cellular detection and overall cell distribution [27]. Identification of cartilage ECM components, specifically glycosaminoglycans within the tissue, was performed using TB, while Safranin O-Fast Green (SO/FG) was used to detect proteoglycans, especially glycosaminoglycans in the cartilage matrix, which was more suited for the specific quantitative determination of the cartilage polyanions [28].

The arthroscopic and histological scores established by the International Cartilage Repair Society (ICRS) aim to assess the quality of cartilage repair. Meanwhile, the ICRS II score signifies an enhancement in reader reproducibility compared to existing histological grading systems for cartilage repair [15]. Two of the studies used the O’Driscoll histological grading scale. The O’Driscoll score, initially developed as the inaugural histological grading system for articular cartilage defects, was applied to evaluate the efficacy of periosteal grafts in treating full-thickness chondral defects in rabbits. This scoring methodology encompasses various parameters, including cellular morphology, Safranin-O staining of the matrix, surface regularity, structural integrity, thickness, bonding to adjacent cartilage, absence of cellular degenerative changes (hypocellularity), chondrocyte clustering, and lack of degenerative alterations in adjacent cartilage [29]. One of the scoring methods used was Mankin’s histological scoring, which provided histological grading of degeneration of cartilage damage [30]. All the scoring methods were supplemented with immunohistochemical staining of Collagen type II.

## 4. Discussion

This systematic review examined five articles reporting on the enhancement of cartilage regeneration in full-thickness cartilage defects using human amnion. A detailed analysis of MSCs derived from human amnion was conducted, including the isolation and characterization of MSCs from amnion, creation of osteochondral defects in vivo, and the histological examination of the cartilage defect. This review can serve as reference for future studies on the workflow analysis of deriving MSCs from human amnion to enhance cartilage for full-thickness cartilage defects.

### 4.1. HAM Scaffolds Enhancing Osteochondral Repair and Cartilage Regeneration

Human amniotic membrane (HAM) scaffolds play a crucial role in osteochondral repair and cartilage regeneration, offering distinct advantages over other biological or synthetic scaffolds. Notably, HAM demonstrates favorable biocompatibility and minimal risk of immunological rejection upon in vivo implantation, owing to its wide availability and lack of ethical controversies. Additionally, HAM is enriched with abundant collagen fibers, including types IV, V, and VII, within its basement membrane, in addition to fibronectin and laminin [30]. These collagen types, particularly type II collagen, which is characteristic of hyaline cartilage, are essential for promoting the formation of hyaline-like cartilage tissues [31].

Studies have demonstrated that HAM scaffolds, with their porous structure and adequate mechanical strength derived from collagen fibers, facilitate cell adhesion and proliferation, particularly for mesenchymal stem cells (MSCs) [32]. In vivo evaluations, complemented by macroscopic and histological assessments, have shown that both HAM alone and HAM combined with MSCs promote cartilage repair. Interestingly, HAM combined with MSCs exhibits superior efficacy in enhancing cartilage repair compared to HAM alone, highlighting the potential synergistic effects between HAM and MSCs. However, further elucidation of the specific mechanisms underlying this enhanced efficacy is warranted.

In addition to cartilage repair, HAM scaffolds have been explored in various tissue engineering applications, including wound repair. Studies have investigated the use of induced MSCs and adipose-derived stem cells seeded onto HAM scaffolds for skin substitutes, demonstrating promising results in wound healing [24].

Moreover, HAM scaffolds offer a novel approach for the treatment of osteochondral defects. While Autologous Chondrocyte Implantation (ACI) is considered the gold standard, its complex surgical procedures and time-consuming chondrocyte expansion in vitro pose challenges. In contrast, HAM scaffolds present a readily available and potentially more efficient alternative for promoting cartilage regeneration.

In summary, the incorporation of HAM scaffolds, particularly in combination with MSCs, holds great promise for enhancing osteochondral repair and cartilage regeneration. Further research into the specific mechanisms and optimization of these approaches is essential for advancing their clinical translation [31].

### 4.2. Bioactive Component of HAM Promoting Tissue Repair and Regeneration

HAM contains a wide range of growth factors and cytokines that work together to support its extraordinary capacity for regeneration [32]. These bioactive compounds are vital in coordinating the cellular reactions necessary for tissue regeneration and repair. Numerous growth factors, including nerve growth factors such as EGF, are present in the amniotic membrane. They stimulate wound epithelialization and improve adhesion, migration, and differentiation of epithelial cells [33]. Another important component of HAM is the high level of platelet-derived growth factor (PDGF). PDGF family members are mesenchymal cell-derived mitogenic factors that are also essential for tissue healing, angiogenesis, and anti-inflammatory reactions [34]. Vascular endothelial growth factor (VEGF) is also detected in HAM. These factors, including cell recruitment and proliferation as well as inflammatory control, are known to be involved in normal wound healing [35]. Additionally, VEGF is critical in promoting angiogenesis, which is necessary to enable the development of new blood vessels required for delivering nutrients and oxygen to tissues that are regenerating. Placental growth factor (PIGF) belongs to the subfamily of VEGF. At every stage of gestation, the placenta expresses PIGF, a crucial molecule in angiogenesis, at a high level [28]. These factors, which include cell recruitment and proliferation as well as inflammatory control, are known to be involved in normal wound healing.

Another important component of HAM is the tissue inhibitors of MMPs (TIMPs) that function as matrix metalloproteinase (MMP) inhibitors, which are important for stimulating cell proliferation in a variety of cell types and may also have anti-apoptotic properties [36]. Furthermore, interleukin 10 (IL-10), which is mostly released by mast cells and macrophages, is a crucial immunoregulatory cytokine that has strong anti-inflammatory properties. On the other hand, interleukin 4 (IL-4) is an inflammatory cytokine. Interestingly, TIMP-2 levels were shown to have increased inside HAM.

Transforming growth factor (TGF)-β and epidermal growth factor (EGF) are secreted by HAM [37]. These two substances are essential for promoting keratinocyte migration. TGF-β is essential for coordinating the keratinocyte Epithelial–Mesenchymal Transition (EMT) during wound re-epithelialization, as it regulates keratinocyte proliferation in normal skin [38]. Nonetheless, in situations involving chronic wounds, an overabundance of TGF-β may result in keratinocyte cell cycle arrest [34], hence, hindering cell migration. On the other hand, HAM secretes a substance called EGF, mostly expressed in basal keratinocytes of the normal epidermis, governing keratinocyte proliferation, differentiation, and migration [39,40]. Following injury, EGF expression is upregulated in keratinocytes neighboring the wound site, further facilitating wound healing processes. Table 7 shows a summary of the advantages and disadvantages of HAM in cartilage regeneration, based on the reviewed articles.

## 5. Conclusions

Our systematic review has demonstrated that HAM has the capability to enhance cartilage regeneration for osteochondral defect repair. HAM possesses the capacity to act as a promising biological scaffold for cartilage regeneration which has good cytocompatibility. However, it is crucial to clarify the mechanism underlying osteochondral repair; thus, further studies are required.

## Figures and Tables

**Table 1:** Search strategy for systematic review process.

Database	Search Strategy
Web of Science	TI (((((((((TI = (Full-thickness cartilage defect)) OR TI = (Cartilage)) OR TI = (Hyaline cartilage)) OR TI = (Articular cartilage)) OR TI = (Osteochondral defect)) OR TI = (Chondral lesion)) OR TI = (Chondral defect)) OR TI = (Articular cartilage defect)) OR TI = (Articular cartilage lesions)) OR TI = (Knee chondral injury) AND ((((((((((TI = (Human amnion)) OR TI = (HAM)) OR TI = (AM)) OR TI = (Amniotic membrane)) OR TI = (Amnion membrane)) OR TI = (Placenta membrane)) OR TI = (Fetus-membrane)) OR TI = (Amniotic tissue)) OR TI = (Placental-driven biomaterials)) OR TI = (Freeze-dried HAM)) OR TI = (Air-dried HAM) AND ((((((TI = (Cartilage regeneration)) OR TI = (Fibrocartilage formation)) OR TI = (Chondrogenesis)) OR TI = (Cartilage tissue repair)) OR TI = (Articular cartilage regeneration)) OR TI = (Hyaline cartilage formation)) OR TI = (Articular cartilage repair) AB (((((((((AB = (full-thickness cartilage defect)) OR AB = (cartilage)) OR AB = (hyaline cartilage)) OR AB = (articular cartilage)) OR AB = (osteochondral defect)) OR AB = (chondral lesion)) OR AB = (chondral defect)) OR AB = (articular cartilage defect)) OR AB = (articular cartilage lesions)) OR AB = (knee chondral injury) AND (((((((((((AB = (human amnion)) OR AB = (HAM))) OR AB = (AM)) OR AB = (amniotic membrane)) OR AB = (amnion membrane)) OR AB = (placenta membrane)) OR AB = (fetus-membrane)) OR AB = (amniotic tissue)) OR AB = (placental-driven biomaterials)) OR AB = (freeze-dried HAM)) OR AB = (air-dried HAM) AND ((((((AB = (cartilage regeneration)) OR AB = (fibrocartilage formation)) OR AB = (chondrogenesis)) OR AB = (cartilage tissue repair)) OR AB = (articular cartilage regeneration)) OR AB = (hyaline cartilage formation)) OR AB = (articular cartilage repair)
Scopus	((TITLE-ABS-KEY (full AND thickness AND cartilage AND defect) OR TITLE-ABS-KEY (cartilage) OR TITLE-ABS-KEY (hyaline AND cartilage) OR TITLE-ABS-KEY (articular AND cartilage) OR TITLE-ABS-KEY (osteochondral AND defect) OR TITLE-ABS-KEY (chondral AND lesion) OR TITLE-ABS-KEY (chondral AND defect) OR TITLE-ABS-KEY (articular AND cartilage AND defect) OR TITLE-ABS-KEY (articular AND cartilage AND lesions) OR TITLE-ABS-KEY (knee AND chondral AND injury))) AND ((TITLE-ABS-KEY (ham) OR TITLE-ABS-KEY (am) OR TITLE-ABS-KEY (amniotic AND membrane) OR TITLE-ABS-KEY (amnion AND membrane) OR TITLE-ABS-KEY (placenta AND membrane) OR TITLE-ABS-KEY (fetus-membrane) OR TITLE-ABS-KEY (amniotic AND tissue) OR TITLE-ABS-KEY (placental-driven AND biomaterials) OR TITLE-ABS-KEY (freeze-dried AND ham) OR TITLE-ABS-KEY (air-dried AND ham))) AND ((TITLE-ABS-KEY (cartilage AND regeneration) OR TITLE-ABS-KEY (fibrocartilage AND formation) OR TITLE-ABS-KEY (chondrogenesis) OR TITLE-ABS-KEY (cartilage AND tissue AND repair) OR TITLE-ABS-KEY (articular AND cartilage AND regeneration) OR TITLE-ABS-KEY (hyaline AND cartilage AND formation) OR TITLE-ABS-KEY (articular AND cartilage AND repair)))
PubMed	Search: “full-thickness cartilage defect”[Title/Abstract] OR “Cartilage”[Title/Abstract] OR “hyaline cartilage”[Title/Abstract] OR “articular cartilage”[Title/Abstract] OR “osteochondral defect”[Title/Abstract] OR “chondral lesion”[Title/Abstract] OR “chondral defect”[Title/Abstract] OR “articular cartilage defect”[Title/Abstract] OR “articular cartilage lesions”[Title/Abstract] OR “knee chondral injury”[Title/Abstract] AND “human amnion”[Title/Abstract] OR “HAM”[Title/Abstract] OR “AM”[Title/Abstract] OR “amniotic membrane”[Title/Abstract] OR “amnion membrane”[Title/Abstract] OR “placenta membrane”[Title/Abstract] OR “Fetus-membrane”[Title/Abstract] OR “amniotic tissue”[Title/Abstract] OR (“Placental-driven”[All Fields] AND “biomaterials”[Title/Abstract]) OR (“Freeze-dried”[All Fields] AND “HAM”[Title/Abstract]) OR (“Air-dried”[All Fields] AND “HAM”[Title/Abstract]) AND “cartilage regeneration”[Title/Abstract] OR “fibrocartilage formation”[Title/Abstract] OR “Chondrogenesis”[Title/Abstract] OR “cartilage tissue repair”[Title/Abstract] OR “articular cartilage regeneration”[Title/Abstract] OR “hyaline cartilage formation”[Title/Abstract] OR “articular cartilage repair”[Title/Abstract]

**Table 2:** Full list of original articles reviewed (*n* = 5) and the details.

References	Article Title	Journal Details	HAM Used
[11]	Human amniotic membrane as a delivery matrix for articular cartilage repair	Tissue Engineering, Vol. 13, Issue 4, Pages 693–702.	the epithelial side of intact HAM (IHE), basement side of denuded HAM (DHB), and stromal side of denuded HAM (DHS).
[12]	Study of human acellular amniotic membrane loading bone marrow MSCs in repair of articular cartilage defect in rabbits	Genetics and Molecular Research, Vol. 13, Issue 3, Pages 7992–8001.	human acellular amniotic membrane
[13]	Human amniotic mesenchymal stem cell sheets encapsulating cartilage particles facilitate repair of rabbit osteochondral defects	American Journal of Sports Medicine, Vol. 48, Issue 3, Pages 599–611.	hAMSC sheets were constructed with passage 3 hAMSCs.
[14]	hAMSC sheet promotes repair of rabbit osteochondral defects	Stem Cells International, 3967722	chondrogenically induced hAMSC sheet
[15]	Human acellular amniotic membrane scaffolds encapsulating juvenile cartilage fragments accelerate the repair of rabbit osteochondral defects	Bone & Joint Research, Vol. 11, Issue 6, Pages 349–361.	HAAM scaffold

**Table 3:** HAM strategies to promote cartilage regeneration for full-thickness cartilage repair.

References	HAM Origin	HAM Preservation Methods	HAM Deposition	HAM Transplantation	HAM Uses
[11]	Human	Fresh De-epithelialized	On the defect	Rabbit	Seeded with cells
[12]	Human	Fresh	On the defect	Rabbit	Seeded with cells
[13]	Human	Fresh	Into the defect	Rabbit	Sheets seeded with cells
[14]	Human	Fresh	Into the defect	Rabbit	Sheets seeded with cells
[15]	Human	Decellularized	Into the defect	Rabbit	Scaffold seeded with cells

**Table 4:** Isolation method and culture condition of MSCs.

References	Culture Condition	Isolation Method	Generation Used	Characterization
[11]	LG-DMEM/F12 medium containing 10% fetal bovine serum (FBS), 1% penicillin/streptomycin, 1% L-glutamine, and 1% nonessential amino acids	Articular cartilages were harvested from the knee joints of three 2-week-old New Zealand white rabbits	P3	Osteogenic differentiation (alizalin red staining) and chondrogenic differentiation (alcian blue staining)
[12]	-	BMSCs were isolated using the whole bone marrow culture method from rabbit	P3	Cell morphology, adhesion to plastic and superficial markers expression with flow cytometry
[13]	-	hAMSCs were isolated from the amnion of human placentas from 8 full-term births within 6 h.	P3	Chondrogenic differentiation
[14]	LG-DMEM/F12 medium containing 10% fetal bovine serum (FBS), 1% penicillin/streptomycin, 1% L-glutamine, 1% nonessential amino acids, and 5 ng/mL basic fibroblast growth factor PeproTech	hAMSCs were isolated from the amnion of human placentas	P3	Osteogenic differentiation (alzalin red staining) and chondrogenic differentiation (alcian blue staining)
[15]	LG-DMEM/F12 medium containing 10% fetal bovine serum (FBS), 1% penicillin/streptomycin, 1% L-glutamine, and 1% nonessential amino acids was used to culture the hAMSCs.	hAMSCs were isolated from the amnions of human placentas	P3	Osteogenic differentiation (alzalin red staining) and chondrogenic differentiation (alcian blue staining)

**Table 5:** Creation of osteochondral defects.

References	Animals (No. Per Condition	Model: Defect Localization and Size	Treatments	Evaluation Methodology	Results
[11]	Rabbit (*n* = 12)	Femoral condyle (5 mm)	Group A: null Group B: control—covered with denuded HAM Group B: denuded HAM + seeded cells	Histology	The defect area was barely filled in the defect-only group or filled with immature cartilage tissue in the denuded HAM group. The defect area was completely filled with fully mature cartilage in the DHS group at 8 weeks.
[12]	Rabbit (*n* = 24)	Bilateral femoral condyle (Diameter 4 mm × depth 3 mm)	Group A: HAAM + BMSCS Group B: HAAM	Microscopic observation Histology	The repair effect in group A was better than in group B, suggesting that in vitro-amplified BMSC had a dominant effect on cartilage defect repair.
[13]	Rabbit (*n* = 24)	Osteochondral defects (Diameter 3.5 mm × depth 3 mm)	Group A: Fibrin glue Group B: hAMSC sheet Group C: cartilage particles Group D: hAMSC sheet/cartilage particles	Macroscopic observation Histology	In the hAMSC sheet/cartilage particle group, the defect area was almost completely filled with a white semitranslucent tissue at 3 months postoperatively, and the boundary connected to the normal cartilage tissue had almost completely disappeared.
[14]	Rabbit (*n* = 15)	Osteochondral defects (Diameter 3.5 mm × depth 3 mm)	Group A: control Group B: noninduced cell sheet Group C: chondrogenically induced cell sheet	Histology	Chondrogenically induced cell sheet group had the better repair effect than the noninduced cell sheet group and the control group.
[15]	Rabbit (*n* = 20)	Osteochondral defects (Diameter 3.5 mm × depth 3 mm)	Group A: control Group B: HAAM scaffold Group C: JCFs Group D: HAAM + JCFs	Histology	HAAM + JCFs has newly formed cartilage fillings in the defects and also found smooth and continuous cartilage.

HAAM—human acellular amniotic membrane; JCFs—juvenile cartilage fragments; BMSCS—bone marrow MSCs.

**Table 6:** Histological examination.

References	Specimen Preparation	Sagittal Sections	Staining	Scoring	Subject to
[11]	10% buffered formalin for 48 h and embedded in paraffin	4 μm	Masson’s trichrome and hematoxylin and eosin (H&E) staining.	ICRS scores	Immunohistochemical staining of Col-II
[12]	Not mentioned	Not mentioned	H&E	Wakitani scoring method	Not mentioned
[13]	Fixed in 4% paraformaldehyde	5 μm	H&E, and Safranin-O/Fast-green (SO/FG)	O’Driscoll histological grading scale	Immunohistochemical staining of Col-II
[14]	4% paraformaldehyde for 24 h and decalcified for approximate 2 weeks. Then, they were embedded in paraffin for histological sectioning	5 μm	H&E, toluidine Blue (TB), Safranin-O/Fast-green (SO/FG)	O’Driscoll histological grading scale and the modified Mankin’s histological scoring	Immunohistochemical staining of Col-I and Col-II
[15]	fixed in 4% paraformaldehyde for 24 h, decalcified for two weeks, and embedded in paraffin for routine histological sectioning	5 μm	H&E, Safranin O/Fast Green (SO/FG), and toluidine blue (TB)	ICRS II scores	Immunohistochemical staining of Col-II

**Table 7:** Advantages and disadvantages of HAM in cartilage regeneration.

Advantages	Disadvantages
HAM demonstrates favorable biocompatibility and minimal risk of immunological rejection upon in vivo implantation.	Cost considerations may hinder the widespread adoption of HAM scaffolds compared to laser-based methods.
HAM is enriched with collagen fibers, including types IV, V, and VII, within its basement membrane, which are essential for promoting the formation of hyaline-like cartilage tissues.	Rigorous clinical validation and large-scale trials are essential to confirm the safety, efficacy, and long-term outcomes of HAM-based cartilage regeneration.
HAM scaffolds, with their porous structure and adequate mechanical strength derived from collagen fibers, facilitate cell adhesion and proliferation, particularly for mesenchymal stem cells (MSCs).	More research is needed to understand how HAM works, especially when combined with MSCs, to improve treatment outcomes and ensure success over the long term.
HAM combined with MSCs exhibits superior efficacy in enhancing cartilage repair compared to HAM alone, highlighting potential synergistic effects between HAM and MSCs.	Utilizing HAM scaffolds for cartilage regeneration may pose regulatory challenges due to variations in sourcing, processing, and standardization of the material, which could affect reproducibility and clinical translation.
HAM presents a readily available alternative for promoting cartilage regeneration, offering a novel approach for the treatment of osteochondral defects compared to complex surgical procedures like Autologous Chondrocyte Implantation (ACI).	

## Data Availability

The original contributions presented in the study are included in the article, further inquiries can be directed to the corresponding authors.
